# Association of the triglyceride–glucose index with albuminuria: a systematic review and meta-analysis

**DOI:** 10.3389/fmed.2026.1798030

**Published:** 2026-07-03

**Authors:** Ying-Yan Li, Chun-Feng Deng, Yi Zeng, Xi Liu

**Affiliations:** Longgang Central Hospital of Shenzhen, Shenzhen, Guangdong, China

**Keywords:** albuminuria, insulin resistance, meta-analysis, systematic review, triglyceride–glucose index

## Abstract

**Background:**

Albuminuria is an early marker of glomerular microvascular injury and can predict the progression of chronic kidney disease (CKD). The triglyceride-glucose (TyG) index, a convenient surrogate indicator of insulin resistance, has been associated with renal injury, but its relationship with albuminuria remains unclear. This study aims to systematically evaluate the relationship between the TyG index and the risk of albuminuria.

**Methods:**

We searched PubMed, Embase, Web of Science, and the Cochrane Library for relevant studies published up to January 1, 2026. Observational studies reporting the association between the TyG index and albuminuria, and providing effect estimates with 95% confidence intervals (CIs) were included. Study quality was assessed using the Newcastle–Ottawa Scale (NOS) and the Agency for Healthcare Research and Quality (AHRQ) tool. Heterogeneity was assessed using the *I*^2^ statistic, and subgroup analyses, sensitivity analyses, and publication bias assessments were conducted.

**Results:**

A total of 14 studies (13 cross-sectional studies and 1 cohort study) were included, with a combined sample size of 59,148 participants. The cross-sectional pooled analysis showed that a higher TyG index was significantly associated with albuminuria (OR = 2.37, 95% CI: 1.26–4.43; *I*^2^ = 85.5%). After excluding one outlier study reporting an extreme effect size, the association remained statistically significant (OR = 1.61, 95% CI: 1.48–1.75), with heterogeneity decreasing to 53.5%. Subgroup analyses by population type, region, adjustment for renal function indicators, sample size, and BMI adjustment revealed consistent positive correlations without significant intergroup differences. Leave-one-out analyses demonstrated robust results. The results of the publication bias risk assessment were primarily driven by an outlier study. After excluding that study, no significant small-study effects were observed. The sole cohort study suggested an increased risk of new-onset albuminuria with higher TyG index (HR = 1.19, 95% CI 1.03–1.37).

**Conclusion:**

Available evidence indicates that elevated TyG index correlates with increased risk of albuminuria across different populations. Limited longitudinal evidence also supports an association between TyG and heightened risk of new-onset albuminuria. The TyG index may serve as a simple metabolic marker for early renal risk stratification, though further large-scale prospective studies are needed to clarify temporal relationships and potential causality.

**Systematic review registration:**

https://www.crd.york.ac.uk/PROSPERO/view/CRD420261290210, identifier CRD420261290210.

## Introduction

Chronic kidney disease (CKD) is one of the major global public health issues today, having become a significant cause of mortality and disease burden, with a prevalence exceeding 10% in the general population worldwide (1). It is worth noting that early-stage kidney injury typically lacks obvious clinical symptoms and is often detected only after structural damage or functional decline has occurred (2, 3). Identifying sensitive early markers of renal injury is crucial for enabling timely intervention. Among existing biomarkers, albuminuria is widely recognized as one of the earliest and most sensitive indicators of glomerular microvascular injury, while also serving as an important predictor of CKD progression and cardiovascular events (4, 5). More importantly, even when estimated glomerular filtration rate (eGFR) is normal, the presence of albuminuria indicates potential early renal impairment (6). Therefore, identifying modifiable metabolic risk factors associated with albuminuria holds significant clinical importance for achieving early risk stratification and preventing adverse renal outcomes.

Insulin resistance (IR) and metabolic disorders are the core drivers of early microvascular damage in the kidneys (7). The hyperinsulinemia associated with IR can cause glomerular hyperfiltration and increased vascular permeability, while the chronic low-grade inflammation and vascular endothelial dysfunction associated with IR further compromise the glomerular filtration barrier (7). It is noteworthy that IR is consistently associated with an increased risk of albuminuria in both diabetic and non-diabetic populations. A cohort study found that lower insulin sensitivity prior to the onset of type 2 diabetes mellitus (T2DM) was associated with an increased risk of albuminuria after the onset of diabetes (8). Moreover, interventions targeting metabolic dysfunction and IR may help reduce albuminuria or slow renal risk progression, further supporting the pathogenic role of IR in early renal injury (9). Therefore, IR caused by metabolic abnormalities may be one of the key mechanisms triggering early kidney damage (10).

The triglyceride-glucose (TyG) index is a metric calculated from fasting triglycerides and fasting blood glucose, widely regarded as a convenient surrogate marker for assessing IR (11, 12). Studies have shown that the TyG index is highly correlated with the results of the hyperinsulinemic–euglycemic clamp, the gold standard for IR, with favorable sensitivity and specificity (13, 14). Compared to HOMA-IR or clamp tests, the TyG index serves as a convenient and cost-effective alternative indicator for IR. It demonstrates comparable or even superior performance to traditional methods in identifying IR and metabolic syndrome (15). Given the central role of IR in renal microvascular injury, researchers have increasingly focused on the relationship between the TyG index and renal outcomes in recent years. Accumulating evidence indicates that an elevated TyG index is significantly associated with renal injury and adverse renal events. A meta-analysis involving nearly 59,000 adults revealed that an elevated baseline TyG index is associated with a 44% increased risk of developing new-onset CKD (HR 1.44, 95% CI:1.33–1.56) (16). Additionally, among individuals with confirmed CKD, the systematic review revealed that patients with elevated TyG index exhibited a significantly increased risk of further renal function decline and progression to end-stage renal disease (17). In summary, these studies suggest that the TyG index is closely associated with renal metabolic stress and adverse renal outcomes, spanning the entire process of CKD development and progression.

However, existing research has primarily focused on CKD outcomes defined by eGFR decline or composite renal endpoints, which represent relatively advanced stages of kidney injury (18, 19). In contrast, albuminuria reflects earlier stages of glomerular injury, typically preceding eGFR decline (3). Although multiple observational studies have reported associations between the TyG index and albuminuria (6, 14, 20), effect sizes remain inconsistent, and no systematic review or meta-analysis has comprehensively quantified this relationship. Clarifying this association is crucial for determining whether the TyG index can serve as a metabolic biomarker for early glomerular microvascular injury.

In this study, the authors conducted a systematic review and meta-analysis to comprehensively evaluate the association between the TyG index and albuminuria. The aim was to clarify the consistency and robustness of existing evidence and assess whether the TyG index could serve as a simple, practical tool for early identification and risk stratification of individuals at high risk for kidney disease.

## Methods

### Registration of review protocol

The present meta-analysis adhered to the PRISMA reporting standards (21) and was prospectively registered in PROSPERO (Registration No. CRD420261290210). The PRISMA 2020 checklist is provided in Supplementary Table 1.

### Search strategy

This study conducted a systematic literature search across four electronic databases: Web of Science, PubMed, Embase, and Cochrane Library. The search period spanned from the inception of each database up to January 1, 2026. Search terms included those related to the TyG index and albuminuria, such as “triglyceride–glucose index,” “TyG index,” “albuminuria,” and “microalbuminuria.” Detailed search strategies for each database are provided in Supplementary Table 2.

### Inclusion and exclusion criteria

Inclusion criteria are as follows:

(1) Observational studies (cross-sectional, cohort, or case-control studies) reporting associations between the TyG index and albuminuria. (2) Studies defining albuminuria using urinary albumin-related indicators, including urinary albumin-to-creatinine ratio (UACR) ≥30 mg/g or urinary albumin excretion ≥30 mg/24 h (2). (3) Studies reporting odds ratios (OR), risk ratios (RR), or hazard ratios (HR) with 95% confidence intervals (CIs). (4) Studies involving human subjects only.

Exclusion criteria are as follows:

(1) Studies that did not report the target outcome or for which effect size data could not be obtained. (2) Duplicate publications. (3) Literature lacking original data, such as reviews, conference abstracts, editorials, guidelines, or case reports. (4) Studies whose included populations overlapped with those already included in the analysis.

### TyG definition

All studies included in this analysis employed the TyG index as a representative indicator for IR. The TyG index is commonly defined as (22): TyG = ln [fasting triglycerides (mg/dL) × fasting glucose (mg/dL)/2]. Effect size extraction adhered to the original studies’ exposure definitions. When a single study provided multiple models with varying degrees of adjustment, the most fully adjusted model was prioritized for inclusion.

### Data extraction

Two researchers (Y-YL. and C-FD) independently extracted and cross-checked the data from studies included in the meta-analysis. Disagreements regarding study inclusion or data extraction were resolved through discussion and consultation with a third researcher (YZ). Information extracted from each study included: publication year, country, study design, study population, sample size, mean or median age, proportion of males, follow-up duration (for cohort studies), diagnostic criteria for albuminuria, reported effect measures (OR, HR, or RR), and covariates adjusted for in multivariate regression models.

### Quality assessment

Two researchers (Y-YL and C-FD) independently assessed the methodological quality of included studies. Cohort studies were assessed using the Newcastle-Ottawa Scale (NOS) (23, 24), while cross-sectional studies were evaluated according to the Agency for Healthcare Research and Quality (AHRQ) criteria (25). Studies scoring below 5 points were deemed low quality, 5–7 points indicated moderate quality, and 8 points or above represented high quality. Disagreements arising during quality assessment were resolved through discussion and consultation with a third senior researcher (YZ).

### Certainty assessment

The certainty of evidence for the main outcomes was assessed using the Grading of Recommendations Assessment, Development and Evaluation (GRADE) framework. The assessment of evidence certainty covered dimensions such as risk of bias, inconsistency, indirectness, imprecision, and publication bias. Since all included studies were observational, the initial certainty of evidence was rated as low, and the certainty grade was downgraded when serious issues were identified in the aforementioned dimensions.

### Statistical analyses

Cross-sectional studies used OR as effect measures, while cohort studies employed HR, with corresponding 95% CIs. When studies reported multiple effect estimates with varying degrees of adjustment, the model with the most comprehensive adjustment for confounders was prioritized for meta-analysis. Heterogeneity across studies was assessed using the *I*^2^ statistic. A random-effects model was applied when significant heterogeneity existed (*I*^2^ ≥ 50%), a fixed-effects model was used when heterogeneity was low (*I*^2^ < 50%). *I*^2^ values indicated low heterogeneity (<25%), moderate heterogeneity (25–75%), and high heterogeneity (>75%). To explore potential sources of heterogeneity and assess result robustness, subgroup analyses were conducted based on population characteristics, region, adjustment for renal function, sample size, and body mass index (BMI) adjustment. Sensitivity analysis using the leave-one-out method evaluated the impact of individual studies on pooled results. When a study was found to have a significant statistical impact, additional sensitivity analyses were conducted both with and without that study to assess the robustness of the pooled results. Publication bias was visually assessed using funnel plots and quantitatively evaluated with Egger’s regression test and Begg’s rank correlation test when ≥10 studies were included (26, 27). All statistical analyses were performed using R software (version 4.3.2, “meta” and “metafor” packages). A two-sided *P-*value < 0.05 was considered statistically significant.

## Results

### Literature search

**FIGURE 1:**
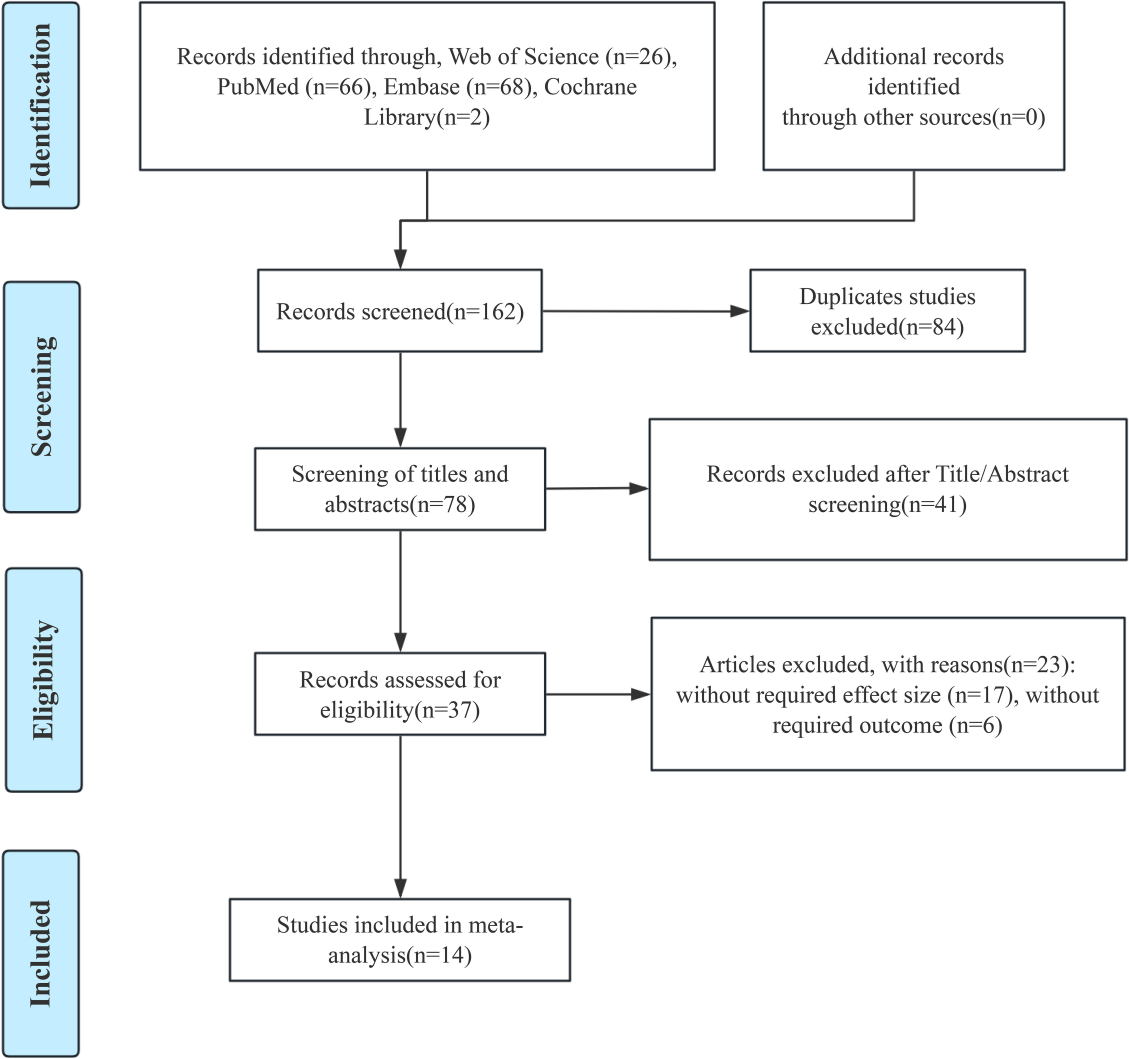
Flow diagram of study selection.

Figure 1 illustrates the literature screening flowchart. A systematic search across Web of Science (*n* = 26), PubMed (*n* = 66), Embase (*n* = 68), and Cochrane Library (*n* = 2) yielded 162 literature records, with no additional sources identified through other channels. After removing 84 duplicate articles, 78 articles proceeded to title and abstract screening. Following the initial screening, 41 studies irrelevant to the research topic were excluded, leaving 37 articles for full-text evaluation. During the full-text review, 23 studies were excluded: 17 failed to provide the required effect size data, and 6 did not report the required outcome measures. Ultimately, 14 studies were included in this meta-analysis.

### Study characteristics

This study included 14 publications (20, 28–40), comprising 13 cross-sectional studies and 1 cohort study, involving a total of 59,148 participants. Among the cross-sectional studies, 6 focused on individuals with T2DM, 6 on the general population, and 1 on individuals with hypertension. The cohort study was conducted in a T2DM population without albuminuria at baseline. Regarding geographic distribution, 8 studies originated from China, while the remaining studies were conducted in the United States and Canada, South Korea, Iran, India and Turkey. Cross-sectional study sample sizes ranged from 570 to 18,087 participants, with mean or median ages spanning 30.7–64.0 years and male proportions ranging from 30.2 to 61.9%. All studies defined albuminuria using the urinary albumin-to-creatinine ratio (UACR ≥30 mg/g) or equivalent urinary albumin excretion criteria. Cohort studies defined new-onset albuminuria as the development of UACR ≥30 mg/g during follow-up. All studies reported multivariable-adjusted effect values, with common adjustment factors including age, sex, body mass index, blood pressure, lipid profile, glycemic indicators, and renal function parameters. Detailed characteristics of each study are presented in Table 1.

**TABLE 1:** Characteristics of the studies included in the quantitative and qualitative review.

First author, reference	Country	Study design	Population	Sample size	Mean age (years)	Male sex (%)	Median follow-up time (years)	Diagnosis of albuminuria	Covariate adjustments	NOS score	AHRQ score
Yilmaz and Erinc (28)	Turkey	Cross-sectional	T2DM population	570	59 (52–66)	47.9%	/	UACR ≥30 mg/g	Hyperlipidemia and eGFR	/	9
Liu et al. (29)	China	Cross-sectional	T2DM population	938	55.59 ± 13.84	61.90%	/	UACR ≥30 mg/g	Age, sex, BMI, duration of diabetes, SBP, DBP, HbA1c, LDL-C, HDL-C, eGFR, hypertension, coronary heart disease, smoking, and drinking	/	8
Sun et al. (31)	China	Cross-sectional	General population	1,021	61.0 (53, 70)	48.70%	/	UACR ≥30 mg/g	Age, sex, smoking, alcohol drinking, BMI, lipid-lowering drug use, total cholesterol, serum uric acid, ALT, AST, hypertension	/	8
Li et al. (32)	United States and Canada	Cross-sectional	General population	18,087	48.93 ± 18.24	48.54%	/	UACR ≥30 mg/g	Gender, age, race, BMI, waist circumference, education level, smoking, alcohol drinking, SBP, DBP, AST, ALT, serum uric acid, total cholesterol, LDL-C, HDL-C, serum total calcium, hypertension, and diabetes status	/	9
Tian et al. (33)	China	Cross-sectional	Hypertension population	789	Median 62.0 years (IQR 55.0–67.0)	52.10%	/	UACR ≥30 mg/g	Age, sex, current cigarette smoking, current alcohol consumption, BMI, SBP, DBP, HDL-C, LDL-C, uric acid, and duration of diagnosed hypertension	/	7
Nabipoorashrafi et al. (20)	Iran	Cross-sectional	T2DM population	2,934	56.46 ± 10.61	47.10%	/	Persistent albuminuria >30 mg/day, confirmed on two measurements 3–6 months apart	Age, sex, duration of diabetes, SBP, DBP, waist circumference, retinopathy, lipid-lowering agent use, LDL-C, and HOMA-IR	/	9
Du et al. (34)	China	Cross-sectional	General population	1,733	43 Years (median 40–45)	56.60%	/	UACR ≥30 mg/g	Age, sex, SBP, serum creatinine, physical activity, smoking habit, and alcohol consumption	/	9
Oh et al. (35)	South Korea	Cross-sectional	General population	5,420	30.7 ± 6.0	46.40%	/	UACR ≥30 mg/g	Age, sex, smoking status, alcohol consumption, education level, income level, BMI, SBP, hemoglobin, eGFR (CKD-EPI), and history of hypertension, diabetes, and dyslipidemia	/	9
Ji et al. (36)	China	Cross-sectional	General population	6,015	62.35 ± 7.60	34.31%	/	UACR ≥30 mg/g	Age, sex, BMI, SBP, DBP, HDL-C, LDL-C, estimated glomerular filtration rate, self-reported coronary heart disease, stroke, antihypertensive drugs, hypoglycemic drugs, and lipid-lowering drugs	/	9
Xu et al. (37)	China	Cross-sectional	General population	3,439	57.0 (52.0–62.0)	30.21%	/	UACR ≥30 mg/g	Sex, age, education level, smoking history, hypertension, family history of diabetes, HbA1c, HDL-C, LDL-C, total cholesterol, 25-hydroxyvitamin D3, BMI, and TG/HDL-C	/	9
Srinivasan et al. (38)	India	Cross-sectional	T2DM population	1,413	56.3 ± 10.0	50.50%	/	Urinary albumin excretion ≥30 mg/24 h Microalbuminuria: 30–300 mg/24 h Macroalbuminuria: >300 mg/ 24 h	Gender, smoking status, duration of diabetes, insulin treatment, SBP, age, and presence of diabetic retinopathy	/	8
Pan et al. (39)	China	Cross-sectional	T2DM population	4,721	59.56 ± 13.02	53.60%	/	UACR ≥30 mg/g	Age, sex, body mass index, and smoking status	/	7
Ou et al. (40)	China	Cross-sectional	T2DM population	1,872	64.0 ± 11.3	43.20%	/	UACR ≥30 mg/g	Age, sex, coronary artery disease, cerebrovascular disease, SBP, DBP, HbA1c, total cholesterol, HDL-cholesterol, LDL-cholesterol, estimated glomerular filtration rate, and use of medications	/	7
Yu et al. (30)	United States and Canada	Cohort study	T2DM population without albuminuria at baseline	10,196	62.8 ± 6.6	61.50%	7	The study defined incident albuminuria as newly developed UACR ≥30 mg/g among participants without albuminuria at baseline.	Age, sex, race, education level, BMI, smoking status, alcohol consumption, SBP, DBP, HbA1c, duration of diabetes, serum creatinine, baseline UACR, use of ACE inhibitors or ARBs	9	/

T2DM, type 2 diabetes mellitus; UACR, urinary albumin-to-creatinine ratio; BMI, body mass index; SBP, systolic blood pressure; DBP, diastolic blood pressure; HbA1c, glycated hemoglobin A1c; LDL-C, low-density lipoprotein cholesterol; HDL-C, high-density lipoprotein cholesterol; eGFR, estimated glomerular filtration rate; ALT, alanine aminotransferase; AST, aspartate aminotransferase; HOMA-IR, homeostasis model assessment of insulin resistance; TG/HDL-C, triglyceride-to-HDL cholesterol ratio; CKD-EPI, chronic kidney disease epidemiology collaboration equation; NOS, Newcastle–Ottawa Scale; AHRQ, Agency for Healthcare Research and Quality; IQR, interquartile range; ACE inhibitors, angiotensin-converting enzyme inhibitors; ARBs, angiotensin II receptor blockers.

### Quality assessment

Cross-sectional studies employed the AHRQ scale for quality assessment, while cohort studies utilized the NOS scale. The AHRQ scores for the 13 cross-sectional studies ranged from 7 to 9 points, with an average score of 8.31. Eleven of these studies scored ≥8 points, indicating overall methodological quality at a medium-to-high level. One cohort study achieved a score of 9 on the NOS scale, indicating high quality. The overall average quality score for all included studies was 8.36. Detailed scoring results for each study are presented in Supplementary Tables 3, 4.

### Cross-sectional study on the association between TyG and albuminuria

**FIGURE 2:**
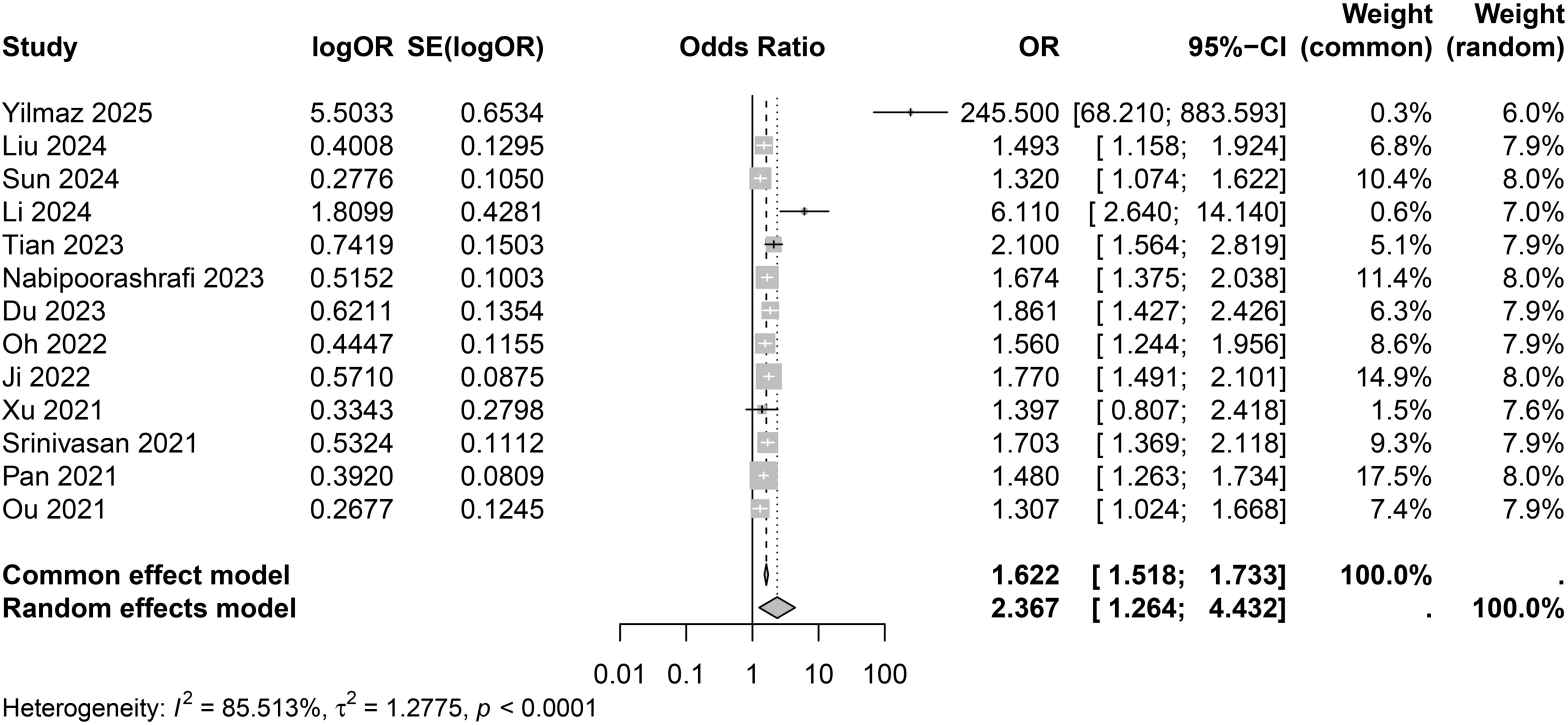
Forest plot of cross-sectional studies including all eligible studies.

**FIGURE 3:**
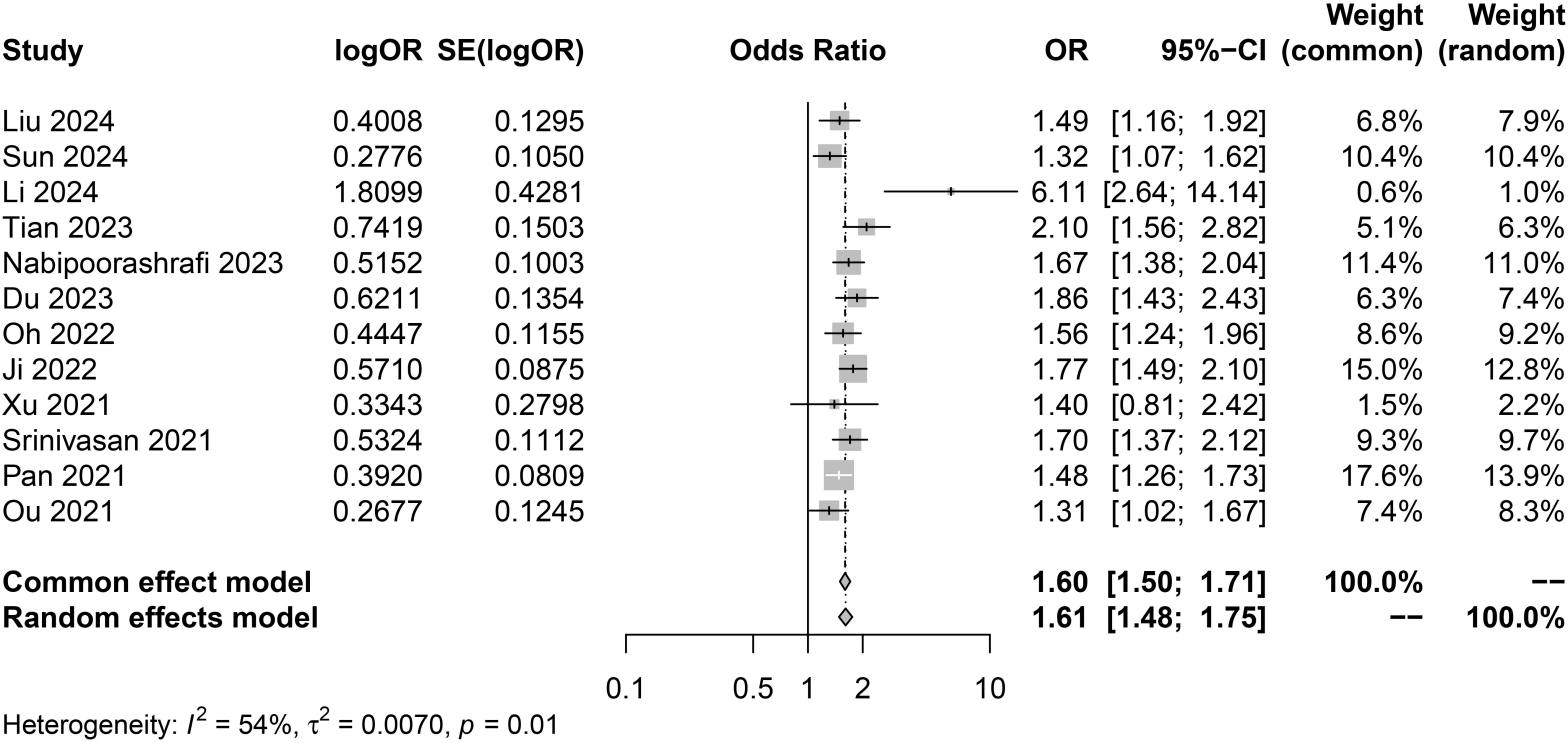
Forest plot of cross-sectional studies after excluding the outlying study.

A total of 13 cross-sectional analyses were included in the pooled analysis, encompassing 48,952 participants. The quantitative meta-analysis revealed a high degree of heterogeneity among studies (*I*^2^ = 85.5%), hence data were pooled using a random-effects model. As shown in Figure 2, the pooled analysis revealed a statistically significant association between higher TyG index levels and the occurrence of albuminuria (OR = 2.37, 95% CI: 1.26–4.43). Among the included studies, the effect size reported by Yilmaz and Erinc (28) was significantly higher than that of the other studies. Therefore we conducted a sensitivity analysis excluding this outlier study with a statistically significant impact to assess the robustness of the pooled results. After excluding the study by Yilmaz and Erinc, the association between the TyG index and albuminuria remained statistically significant (OR = 1.61, 95% CI: 1.48–1.75), and heterogeneity decreased from 85.5 to 53.5%. These results suggest that the overall direction of the association is robust, but the magnitude of the pooled effect size and the degree of heterogeneity among studies were clearly influenced by this outlier study. Figure 3 shows the forest plot of the main meta-analyses of cross-sectional studies after excluding the outlier study.

### Subgroup analysis

**FIGURE 4:**
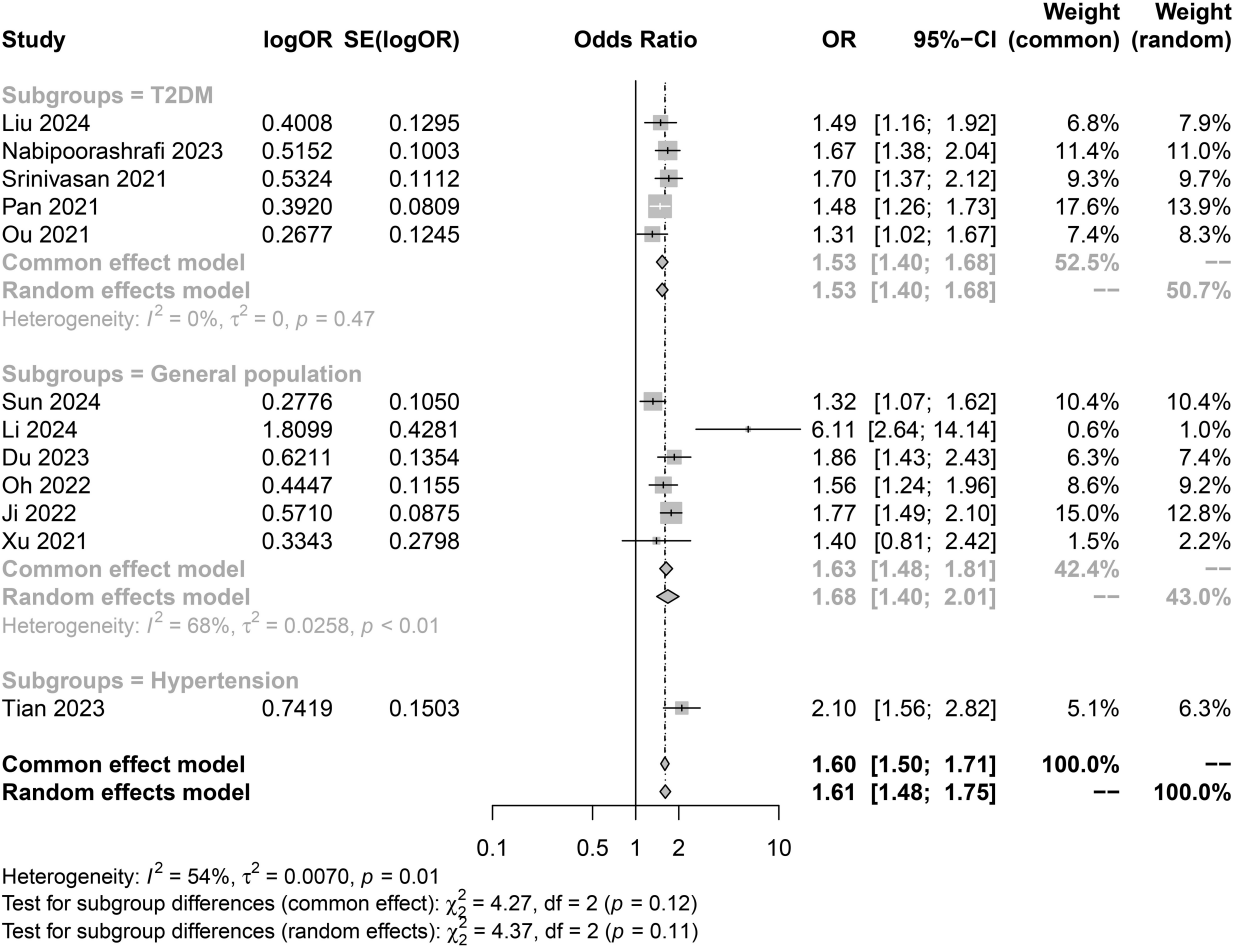
Population subgroup analysis after excluding the outlying study.

To further explore potential sources of heterogeneity and validate the stability of results, subgroup analyses were conducted based on population characteristics, adjusted covariates, region, sample size, and BMI adjustment. Subgroup analyses by population group revealed that the TyG index was positively correlated with albuminuria in both the T2DM population and the general population, as well as in the hypertension population. As shown in Supplementary Figure 1, in the T2DM subgroup, the random-effects model showed a positive but imprecise pooled effect (OR = 3.31, 95% CI: 0.70–15.50), with high heterogeneity (*I*^2^ = 92.1%). In the general population subgroup, this association remained statistically significant (OR = 1.68, 95% CI: 1.40–2.01; *I*^2^ = 68.5%). Only one study was included in the subgroup of individuals with hypertension, which also showed a significant positive association (OR = 2.10, 95% CI: 1.56–2.82). Tests for differences between subgroups did not reach statistical significance, suggesting that population type did not significantly alter the association between the TyG index and albuminuria. After excluding the outlier study by Yilmaz and Erinc, the association remained statistically significant in the T2DM subgroup (OR = 1.53, 95% CI: 1.40–1.68), and heterogeneity decreased from 92.1 to 0% (Figure 4). Differences across subgroups remained non-statistically significant. These results suggest that the association between the TyG index and albuminuria is generally consistent across different populations, and that the high heterogeneity observed in the primary T2DM subgroup was primarily driven by the outlier study by Yilmaz and Erinc.

**FIGURE 5:**
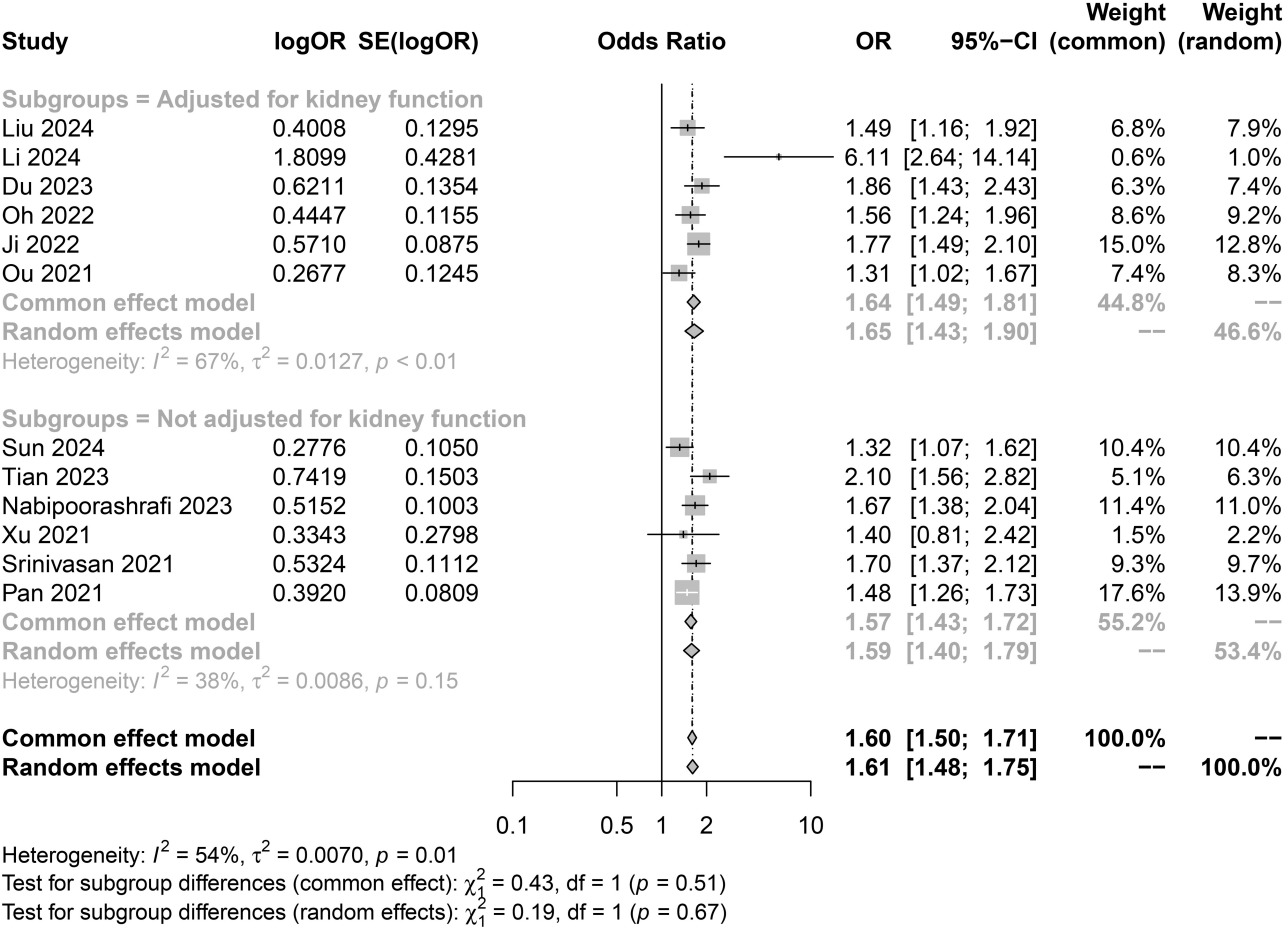
Subgroup analysis according to adjustment for renal function indicators after excluding the outlying study.

To assess the impact of adjusting for renal function on study outcomes, a subgroup analysis was conducted based on whether renal function measures (eGFR, serum creatinine) were adjusted for in the multivariate model. Seven studies adjusted for kidney function indicators as covariates, while other six studies did not adjust for kidney function indicators as covariates. As shown in Supplementary Figure 2, in studies adjusted for renal function, the pooled effect size was positive but of low precision (OR = 3.66, 95% CI: 1.00–13.38), and heterogeneity was high (*I*^2^ = 91.8%). In studies that did not adjust for renal function, the association remained statistically significant (OR = 1.59, 95% CI: 1.40–1.79), with low heterogeneity (*I*^2^ = 38.4%). The test for differences between subgroups did not reach statistical significance, suggesting that adjustment for baseline renal function did not significantly alter the association between the TyG index and albuminuria. After excluding the outlier study by Yilmaz and Erinc, the association in the subgroup adjusted for renal function remained statistically significant (OR = 1.65, 95% CI: 1.43–1.90), and heterogeneity decreased from 91.8 to 67% (Figure 5). Results in the unadjusted subgroup remained largely unchanged, with the difference between subgroups still failing to reach statistical significance. These findings suggest that the positive correlation between the TyG index and albuminuria is generally consistent regardless of renal function adjustment, while the high heterogeneity observed in the adjusted subgroup was partly driven by the outlier study by Yilmaz and Erinc.

**FIGURE 6:**
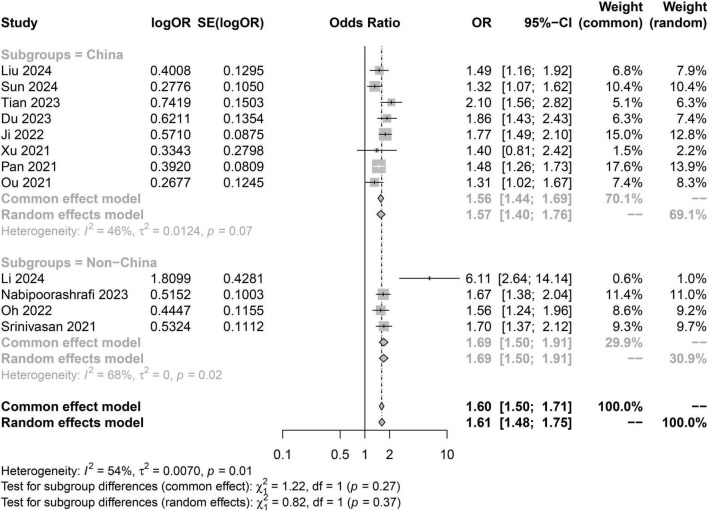
Region subgroup analysis after excluding the outlying study.

Subgroup analyses were conducted based on study region (China and non-China). A total of 8 studies originated from China, while 5 studies were conducted in non-Chinese regions. In both regional subgroups, the TyG index was positively correlated with albuminuria. As shown in Supplementary Figure 3, in the Chinese studies, this association was statistically significant (OR = 1.57, 95% CI: 1.40–1.76), with moderate heterogeneity (*I*^2^ = 45.9%). In non-Chinese studies, the pooled effect was positive but of low precision (OR = 5.43, 95% CI: 0.88–33.43), with high heterogeneity (*I*^2^ = 94%). The test for subgroup differences did not reach statistical significance, suggesting that the study region did not significantly alter the association between the TyG index and albuminuria. After excluding the outlier study by Yilmaz and Erinc, the association in studies from non-Chinese regions became statistically significant (OR = 1.69, 95% CI: 1.50–1.91), and heterogeneity decreased from 94 to 68% (Figure 6). The pooled effect size for studies conducted in China remained largely unchanged, and the test for differences between subgroups still failed to reach statistical significance. These results suggest that the association between the TyG index and albuminuria is generally consistent across different regions, while the high heterogeneity and imprecision of results in the major non-Chinese subgroup were primarily influenced by the outlier study by Yilmaz and Erinc.

**FIGURE 7:**
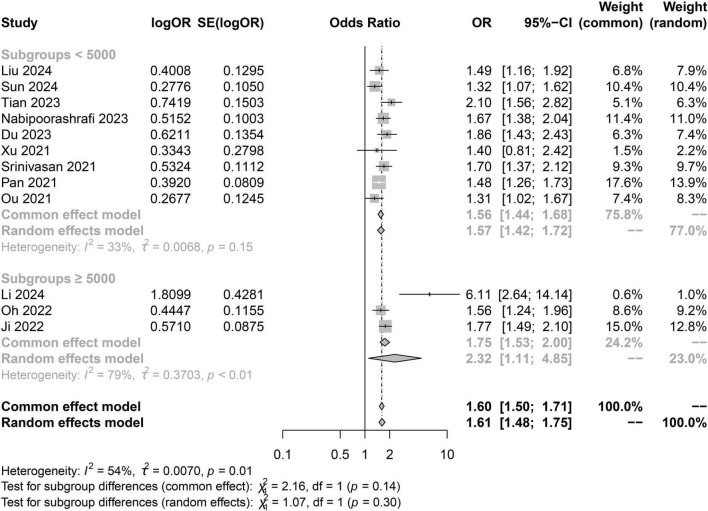
Sample size subgroup analysis after excluding the outlying study.

Subgroup analyses were conducted based on sample size (< 5,000 and ≥5,000 participants). A total of 10 studies had sample sizes < 5,000 participants, while 3 studies had sample sizes ≥5,000 participants. In both subgroups based on sample size, the TyG index was significantly positively correlated with albuminuria. As shown in Supplementary Figure 4, in studies with a sample size <5,000 cases, the pooled effect size was OR = 2.40 (95% CI: 1.03–5.59), with high heterogeneity (*I*^2^ = 87.5%). In studies with a sample size of ≥5,000 cases, the pooled effect size was OR = 2.32 (95% CI: 1.11–4.85), with similarly high heterogeneity (*I*^2^ = 79%). Tests for differences between subgroups did not reach statistical significance, suggesting that sample size did not significantly alter the association between the TyG index and albuminuria. After excluding the study by Yilmaz and Erinc, the association in the subgroup with sample sizes <5,000 remained statistically significant (OR = 1.57, 95% CI: 1.42–1.72), and heterogeneity decreased from 87.5 to 33% (Figure 7). The results for the subgroup with sample sizes ≥5,000 remained unchanged, and the test for differences between subgroups still did not reach statistical significance. These results suggest that the association between the TyG index and albuminuria is generally consistent across subgroups of different sample sizes, while the high heterogeneity observed in the subgroup with <5,000 cases was primarily driven by the outlier study by Yilmaz and Erinc.

**FIGURE 8:**
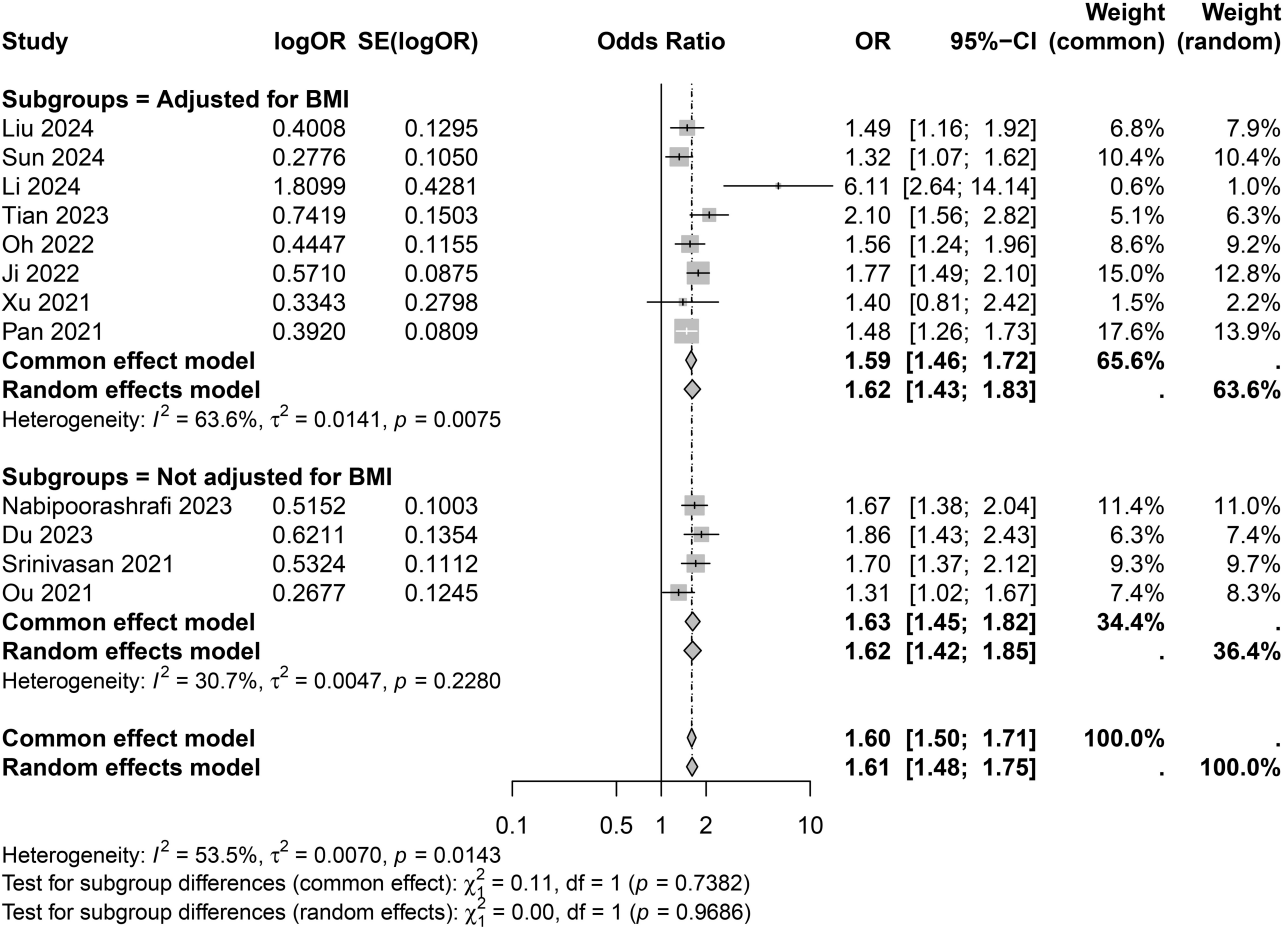
Subgroup analysis according to BMI adjustment after excluding the outlying study.

Since BMI may be an important effect modifier in the association between the TyG index and albuminuria, we further conducted subgroup analyses based on whether BMI was adjusted for in the multivariable models. As shown in Supplementary Figures 5, 8 cross-sectional studies adjusted for BMI, while 5 did not. In studies adjusted for BMI, a higher TyG index was significantly associated with albuminuria (OR = 1.62, 95% CI: 1.43–1.83), with moderate heterogeneity (*I*^2^ = 63.6%). In studies that did not adjust for BMI, the pooled effect remained positive but was imprecisely estimated (OR = 4.13, 95% CI: 0.64–26.74), with high heterogeneity (*I*^2^ = 93.6%). The subgroup analysis showed no statistical significance (*P* = 0.3262), suggesting that adjusting for BMI did not significantly alter the association between the TyG index and albuminuria. Given that the study by Yilmaz and Erinc. reported an extreme effect size, we further conducted a sensitivity subgroup analysis after excluding this outlier study. After exclusion, the association remained statistically significant both in studies adjusted for BMI (OR = 1.62, 95% CI: 1.43–1.83; *I*^2^ = 63.6%) and in studies not adjusted for BMI (OR = 1.62, 95% CI: 1.42–1.85; *I*^2^ = 30.7%) (Figure 8). Subgroup differences remained non-significant (*P* = 0.9686). These results suggest that the positive correlation between the TyG index and albuminuria is generally consistent across studies, regardless of whether BMI was adjusted for.

### Sensitivity analysis

**FIGURE 9:**
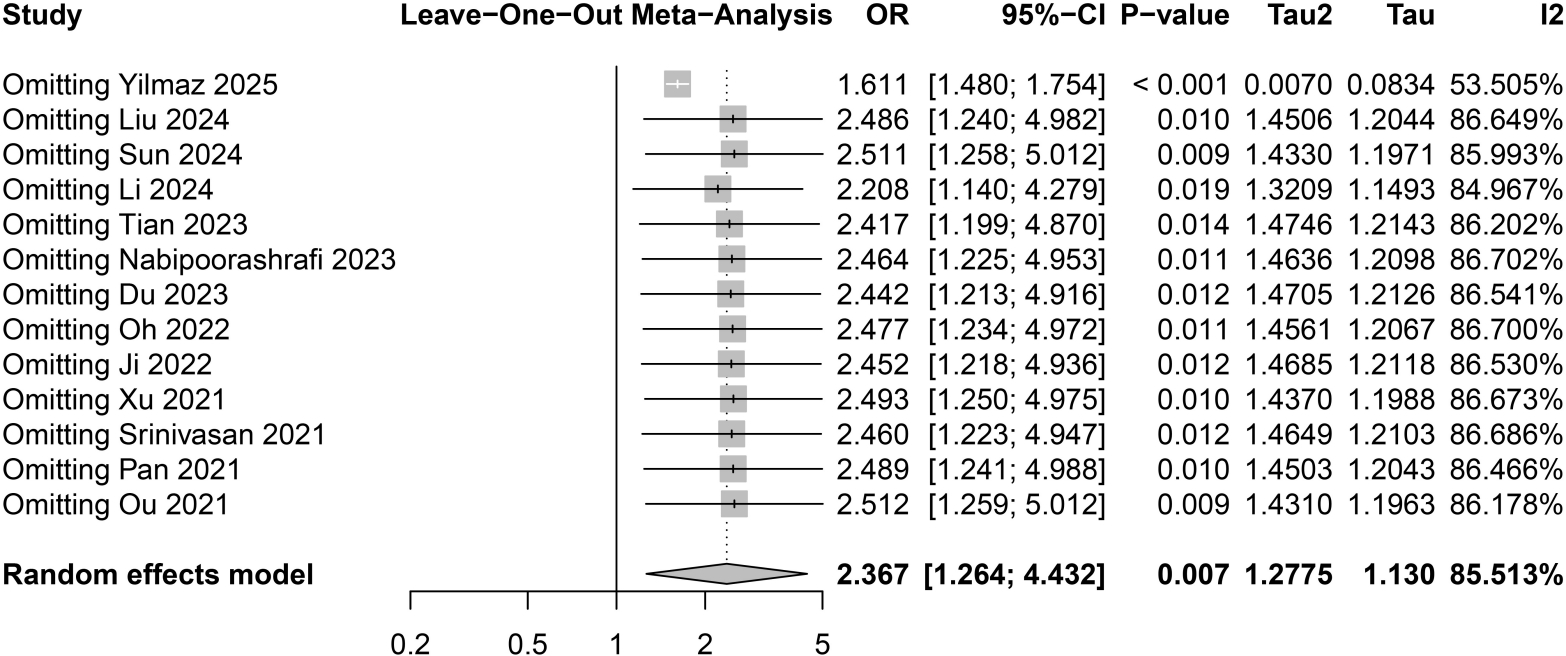
Leave-one-out sensitivity analysis.

Sensitivity analyses were conducted on the cross-sectional study to explore potential sources of heterogeneity and assess the robustness of the findings. In analyses where individual studies were sequentially excluded, the pooled effect size between the TyG index and albuminuria remained stable and statistically significant. The pooled OR ranged from 1.61 (95% CI: 1.48–1.75) to 2.51 (95% CI: 1.26–5.01), indicating no single study exerted a dominant influence on the overall results. However, the study by Yilmaz and Erinc. reported an extreme effect size, which appeared to have a significant impact on the magnitude of the pooled effect size and the heterogeneity among studies. After excluding the study by Yilmaz and Erinc, the pooled OR decreased to 1.61 (95% CI: 1.48–1.75), and heterogeneity decreased from 85.5 to 53.5%. In contrast, excluding any other individual study did not significantly reduce heterogeneity. Sensitivity analysis results for cross-sectional studies are presented in Figure 9.

### Publication bias assessment

**FIGURE 10:**
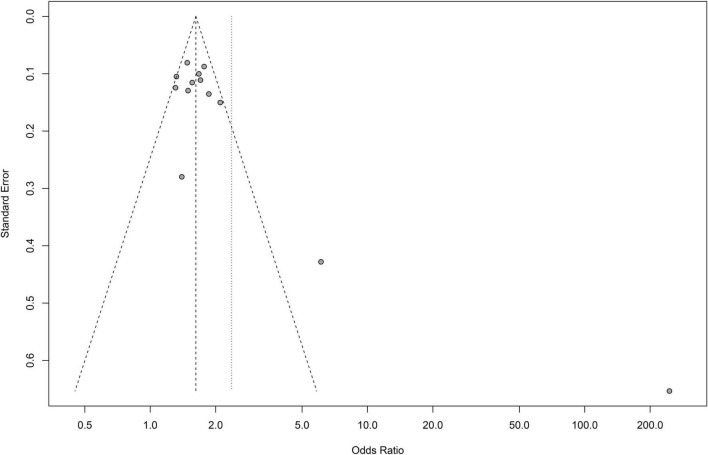
Funnel plot including all eligible cross-sectional studies.

**FIGURE 11:**
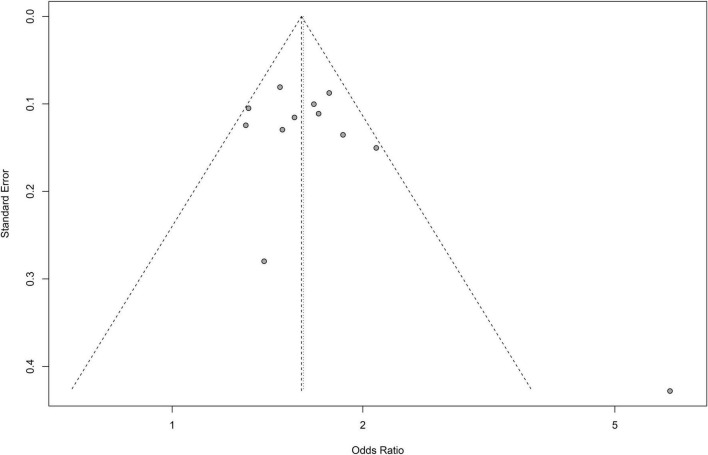
Funnel plot after excluding the outlying study.

To assess potential publication bias, a funnel plot was constructed for the included studies. As shown in Figure 10, the funnel plot exhibits a certain degree of asymmetry, primarily manifested by a single study with an extremely large effect size that deviates significantly from the main body of the funnel plot. The Egger regression test indicates that the asymmetry in the funnel plot is statistically significant (*t* = 3.25, *P* = 0.0077), whereas the Begg’s rank correlation test did not reach statistical significance (*z* = 1.83, *P* = 0.0672). Given the presence of a statistically influential outlier study, we conducted a sensitivity analysis excluding the study by Yilmaz and Erinc. As shown in Figure 11, after excluding this study, the symmetry of the funnel plot improved significantly, and neither the Egger’s test (*t* = 1.66, *P* = 0.1285) nor the Begg’s test (*z* = 1.23, *P* = 0.2171) detected any bias. These results suggest that the asymmetry observed in the funnel plot in the primary analysis was primarily driven by this outlier study, and there is no robust evidence to support the presence of publication bias.

### Cohort study on the association between TyG and albuminuria

Due to the inclusion of only one cohort study, a quantitative meta-analysis of longitudinal evidence could not be performed. This cohort study, conducted in a T2DM population without baseline albuminuria, demonstrated a significant positive association between the TyG index and the risk of new-onset albuminuria. After adjusting for multiple demographic, metabolic, and renal function-related confounders, a higher TyG index remained independently associated with an increased risk of albuminuria development (HR = 1.19, 95% CI: 1.03–1.37). These findings provide preliminary longitudinal evidence supporting the association observed in the cross-sectional analysis of this study.

### GRADE assessment

The results of the GRADE certainty of evidence assessment are summarized in Supplementary Table 5. Based on cross-sectional studies, the certainty of evidence regarding the association between the TyG index and albuminuria was rated as very low. This was primarily due to the observational and cross-sectional designs of the included studies, significant heterogeneity, and the impact of a single outlier study with statistical influence on the pooled effect estimate and the assessment of publication bias. For new-onset albuminuria, the certainty of evidence was also rated as very low, primarily because only one cohort study was available, resulting in limited longitudinal evidence. Overall, although the association between the TyG index and albuminuria is statistically significant, the certainty of evidence remains limited.

## Discussion

This study included 13 cross-sectional studies and 1 cohort study, with a total sample size of 59,148 participants across multiple countries, providing comprehensive evidence for examining the relationship between the TyG index and albuminuria. The results showed that, in the meta-analysis of cross-sectional studies, a higher TyG index was significantly associated with albuminuria (OR = 2.37, 95% CI: 1.26–4.43), although there was considerable heterogeneity among the studies. After excluding the outlier study by Yilmaz and Erinc., this association remained statistically significant (OR = 1.61, 95% CI: 1.48–1.75), and heterogeneity decreased markedly. This association remained consistent across subgroup analyses adjusted for population characteristics, study region, renal function, sample size, and BMI adjustment. In the risk of publication bias assessment, the Egger’s test indicated asymmetry in the funnel plot. However, this result was primarily driven by the outlier study, and the finding was no longer significant after excluding the outlier study. The included cohort study also demonstrated a significant association between elevated TyG index and increased risk of new-onset albuminuria. Although only one cohort study was included, it provided preliminary longitudinal evidence supporting the cross-sectional findings. According to the GRADE assessment, the overall certainty of evidence was very low, indicating that these findings should be interpreted cautiously. Overall, this study provides evidence supporting the association between elevated TyG index and increased albuminuria risk, suggesting that the TyG index may serve as a convenient metabolic marker for identifying and stratifying early kidney injury risk.

Previous meta-analyses have focused on examining the relationship between the TyG index and the risk of CKD. Zhang et al. (16) found a 44% increased risk of new-onset CKD in individuals with high TyG index (RR = 1.44, 95% CI: 1.33–1.56). Ren et al. (19) reported a pooled risk ratio of 1.47 (95% CI: 1.32–1.63) for CKD risk between the highest and lowest TyG quartiles. Sharifi et al. (18) reported a higher pooled effect size (RR = 1.67, 95% CI: 1.51–1.86) in a systematic review encompassing 33 studies. This evidence fully demonstrates the close association between the TyG index and CKD. Recently, Tuo et al. (17) further expanded this evidence, finding that in patients with existing CKD, a high TyG index also significantly increased the risk of CKD progression (HR = 1.52, 95% CI: 1.36–1.70) and end-stage renal disease (ESRD) occurrence (OR = 1.49, 95% CI: 1.12–1.99). However, previous studies have primarily relied on CKD defined by eGFR or composite renal outcomes, which may reflect the mid-to-late stages of renal impairment. In contrast, our study focuses on albuminuria, shifting attention to the earlier stages of kidney injury. Thus, our findings underscore the value of the TyG index, which not only predicts overt CKD but also identifies early kidney injury before measurable declines in eGFR occur. This distinction highlights the potential role of the TyG index as an early warning biomarker throughout the entire progression of kidney disease.

The mechanism underlying the association between the TyG index and albuminuria may be explained by several factors. The TyG index, calculated from fasting blood glucose and triglycerides, is a recognized surrogate marker of IR, which is a key mechanism in early renal injury (41). IR can cause renal dysfunction through multiple pathways, including glomerular hyperfiltration, endothelial dysfunction, and direct damage to podocytes (7, 14). Inflammatory mediators such as IL-6 and TNF-α, elevated in IR states, disrupt glomerular endothelial integrity and increase vascular permeability (6). Concurrently, IR activates the renin-angiotensin-aldosterone system (RAAS), promoting mesangial cell expansion, matrix deposition, and tubulointerstitial fibrosis, thereby facilitating albumin leakage into the urine (14). Additionally, hyperglycemia and high triglycerides, as components of the TyG index, also contribute to kidney damage. Hyperglycemia induces oxidative stress and mitochondrial dysfunction in glomerular cells (42, 43), while high triglycerides lead to lipid deposition in renal tissue, triggering lipotoxicity and inflammatory responses (44). These factors collectively compromise the filtration barrier, potentially inducing albuminuria even before a decline in eGFR occurs. A cross-sectional study found that even after adjusting for traditional IR indicators such as HOMA-IR, the TyG index remained significantly associated with albuminuria (6). This suggests that it reflects not only IR but may also represent a broader state of metabolic disorder. Therefore, the TyG index serves as a sensitive marker for early glomerular injury, holding potential for identifying individuals whose renal function has not yet declined.

This study demonstrates a positive correlation between the TyG index and albuminuria across multiple populations, including those with T2DM, hypertension, and the general population. After excluding the outlying study, the association within the T2DM subgroup became highly consistent (*I*^2^ = 0%), aligning with previous research indicating that IR leads to early renal microvascular damage (7). Conversely, higher heterogeneity was observed in the general population (*I*^2^ = 68%), potentially attributable to variations in age, obesity rates, and other metabolic factors across studies, which may influence the relationship between TyG and albuminuria. Notably, this association remained significant regardless of whether baseline renal function (eGFR, serum creatinine) was adjusted for, suggesting that TyG possesses predictive power for albuminuria even before marked decline in renal function. This supports albuminuria as a predictor of early kidney injury. Furthermore, although this study included only one cohort study, it demonstrated that a higher baseline TyG index was associated with an increased risk of new-onset albuminuria during follow-up, providing preliminary longitudinal evidence for the observed association. However, since most of the included studies were cross-sectional in design, these results should not be interpreted as evidence of causation. Reverse causality and residual confounding remain possible, as albuminuria or early renal dysfunction itself may also be associated with changes in metabolic status, IR, inflammatory responses, and lipid metabolism abnormalities. Therefore, the TyG index should currently be regarded as a potential metabolic marker associated with the risk of albuminuria, rather than a proven causal factor. Well-designed prospective studies, including repeated measurements and adequate adjustment for obesity-related, metabolic, and renal function-related confounders, are still needed in the future.

This study has several limitations. First, most included studies employed cross-sectional designs, limiting the ability to draw causal inferences. Although subgroup analyses adjusted for baseline renal function reduced the possibility of reverse causality, sufficient longitudinal evidence remains lacking. Second, differences in albuminuria measurement methods across studies may have introduced classification errors. Third, although the included studies maximally adjusted for potential covariates that could influence this association, unadjusted potential confounding factors may still exist. Fourth, one of the studies reported an extremely large effect size, which had a significant impact on the pooled effect size and heterogeneity among studies. Although sensitivity analyses showed that the positive correlation between the TyG index and albuminuria remained statistically significant after excluding this outlier study, the pooled effect size should still be interpreted with caution. Fifth, the Egger’s test in the primary analysis indicated asymmetry in the funnel plot. However, this result was primarily driven by a single outlier study with substantial statistical influence, and the finding was no longer significant after excluding that study. Therefore, publication bias or small-study effects cannot be completely ruled out, but there is currently a lack of robust evidence to support their presence. Furthermore, most studies originated from Asia, potentially affecting the generalizability of results to other ethnic populations. Finally, the inclusion of only one high-quality cohort study limits definitive conclusions regarding the causal relationship between the TyG index and albuminuria, suggesting the need for additional prospective research.

Despite these limitations, this study possesses significant strengths. To our knowledge, it represents the first systematic meta-analysis specifically examining the association between the TyG index and albuminuria. By integrating data from nearly 60,000 participants across diverse populations, we achieved comprehensive results. We exclusively included studies reporting multivariate-adjusted risk values to minimize confounding bias. Furthermore, detailed subgroup and sensitivity analyses further validated the consistency and robustness of the findings. Crucially, the association between the TyG index and albuminuria remained significant even after adjusting for renal function, suggesting its potential as an early marker for identifying glomerular damage before eGFR decline. This finding supports the clinical applicability of the TyG index as a simple, accessible tool for early kidney risk screening. Future research should focus on conducting large-scale prospective clinical studies encompassing diverse racial and clinical populations to further validate causal relationships and assess the predictive value of the TyG index across different populations and clinical settings.

## Conclusion

This systematic review and meta-analysis demonstrates that the TyG index exhibits a significant association with albuminuria across diverse populations. This relationship remains consistent regardless of whether baseline renal function is adjusted, suggesting that the TyG index may serve as an early marker of microvascular kidney injury preceding eGFR decline. These findings underscore the potential value of the TyG index as a noninvasive, cost-effective tool for early kidney risk stratification. Future large-scale prospective studies are needed to validate these findings, clarify causal relationships, and assess whether interventions based on the TyG index can help delay the progression of kidney disease.

## Data Availability

The raw data supporting the conclusions of this article will be made available by the authors, without undue reservation.
